# Clinic of Professor Dunglison

**Published:** 1845-05

**Authors:** Samuel G. White

**Affiliations:** Georgia


### CLINICAL LECTURES AND REPORTS.

### PHILADELPHIA HOSPITAL.

*Saturday, January* 11, 1845.

### CLINIC OF PROFESSOR DUNGLISON.

Reported by Dr. Samuel G. White, of Georgia.

Professor Dunglison directed attention, in the commencement of the lecture, to the progress of the cases of JAUNDICE, which were under consideration at the preceding clinic; and which were again exhibited to the class.

One of the patients has greatly improved; every character of the affection, with the exception of slight discoloration of the urine, having disappeared. The bile having resumed its natural course, the skin and tunica conjunctiva have regained their normal appearance, and convalescence is fairly established.

The other case has not proceeded so rapidly, although there is an improvement. The urine continues high coloured, and the yellow tinge of the tissues remains, showing that a considerable quantity of bile still exists in the fluid of the circulation. He has been put on the use of the iodide of mercury, in doses of the one-sixteenth of a grain three times a day, combined with three grains of extract of gentian as a constituent. From the opinion that it has a special action as a "deobstruent" on the abdominal viscera, many practitioners are in the habit of prescribing the extract of taraxacum, instead of that of gentian, in such cases. Whether it have this action, however, the lecturer considers extremely doubtful. He has never seen the slightest evidence of it; nor is it at all probable, that the dandelion possesses the properties so vaguely ascribed to it. It probably has no advantage over the gentian.

A combination of iodine and mercury is peculiarly appropriate in cases like the one in question, where a tonic and eutrophic influence is demanded. When given in large doses it produces ptyalism; but in the minute quantities directed in this case, it will probably have no such effect. The lecturer has found it as easy to affect the mouth with this salt as with any of the preparations of mercury.

In addition to the iodide, a saline has been ordered, with the intention of augmenting the intestinal secretions, and at the same time of exerting a degree of traction downwards on the hepatic ducts. At present, there is slight tenderness over the epigastrium, which may be attributable to the medicines prescribed.

As there probably exists no material organic disease of the liver, this patient will, in all likelihood, recover, although the case may be tedious. The result will be reported on some future occasion.

The lecturer then proceeded to exhibit a case of

### INDURATION OF THE CELLULAR TISSUE.

When the patient—an adult male—was first seen, he presented some peculiarity. He entered the hospital in December last, complaining only of slight cold, with daily fever, and firm tumefaction of the limbs. There was some difficulty in connecting this condition of the cellular tissue with the constitutional disturbance. The professor, however, presuming it to be dependent on organic lesion of some solid viscus, instituted a careful examination, which resulted in the detection of inflammation of the endocardium—ENDOCARDITIS.

The *bruit de soufflet*, or "bellows sound," which is the most prominent physical sign of endocarditis, was well marked, but it has now entirely disappeared. It must be borne in mind, however, that the bellows sound is not always indicative of the actual presence of endocarditis. It may be produced by a narrowing of the aortic or pulmonic, or auriculo-ventricular orifices, or by simple hyperæmia of the endocardium; and, as the lecturer has before remarked, occurs in chlorosis; but the diagnosis of endocarditis may be determined by an investigation of the accompanying functional phenomena.

In addition to the evidences of endocarditis, there were, in this case, others, indicative of PERICARDITIS with liquid effusion. The dulness over the cardiac region was increased in extent, and the sounds of the heart, in consequence of the effusion, appeared to proceed from a distance. These phenomena have now, however, disappeared under the treatment prescribed,—a circumstance which confirms the diagnosis.

When the presence of these cardiac affections was detected, the tumefaction of the limbs became of less moment; yet it was far from being devoid of interest.

Induration of the cellular tissue—*endurcissement du tissu cellulaire*—is very prevalent in the hospitals for sick children abroad, and is often attended with fatal results. In this country it is rarely seen. The cause of its great fatality is not understood, but it is not improbably connected with the organic lesions of the solid organs, that usually accompany and cause it.

Billard is disposed to consider it similar to ordinary dropsy; the only difference being, that the fluid portion of the liquor sanguinis is in this case absorbed, leaving the fibrinous element in the cellular tissue. When the limbs so affected are examined, they are found very resisting, at times, as hard as board. This hardness was well marked in the present case, although, as the class may observe, it has now much diminished.

The lecturer remarked, that in the treatment of this, as of all other affections, the pathological cause should, if possible, be detected, and removed by appropriate measures. From the existence of endo-pericarditis in this case, it became a question, whether it should be treated by active antiphlogistics, disregarding the existing anæmic condition of the system, or whether an opposite plan might not be more judicious. When such a case first presents itself, cups may be applied over the cardiac region, or venesection may even be advisable; but generally it soon becomes necessary to have recourse to less active measures; and manifest advantage accrues from the use of the sulphate of quinia as in rheumatism; these inflammations of the heart, especially in those of broken down frames, being frequently connected with a neuropathic condition of the system, like that which exists in rheumatism, and demanding an analogous treatment.

In the present case, the preliminary use of depletives was unnecessary, as the effusion indicated, that the inflammation had terminated; and, accordingly, the treatment by quinia was at once instituted. Under its employment the patient has improved astonishingly;—the signs of endocarditis and of pericarditic effusion having entirely disappeared.

The lecturer here remarked, that it is by no means easy, at all times, to detect the existence of pericarditis or of endocarditis. The phenomena have, indeed, been so latent, as to pass away entirely without the disease having been suspected; whilst at other times some functional expression has directed the attention of the physician to the heart,—as dropsical effusion, or tumefaction of the limbs, like that in the present instance. Not unfrequently, again, it has happened, that an inflammation of the heart has passed through its whole course without the functional disturbance having attracted the observation of the practitioner to that organ; and dissection alone has revealed the true nature of the lesion. In such cases, the physical signs occasionally afford invaluable aid; yet in pericarditis they may be so obscure as not to lead to any definite diagnosis.

The patient under consideration is manifestly in a cachectic condition; and it may be best to send him away from the wards into the open air, whilst the tonic plan of treatment is continued.

An inference from the happy results in this case would be, that there need be little hesitation, in such cases, in the administration of the sulphate of quinia, where there are no positive special contraindications. It must be borne in mind, that the sulphate of quinia is not a powerful excitant; but rather, as regarded by the Germans, a tonico-paregoric, since it may be adapted for cases where excitants proper might be injurious.

The next case to which the attention of the class was directed, was that of a female labouring under a nervous affection, characterized essentially by

### TREMORS.

When the symptoms first presented themselves they seemed to be periodical, and were supposed to indicate intermittent fever; but, on examination, there were no positive evidences of intermission—no marked cold, or hot, or sweating stage.

At present, there are no very prominent phenomena of any kind; and she is introduced before the class, the professor remarked, merely to show the difficulty that occasionally exists in arriving at any exact conclusion, either as regards diagnosis or therapeutics. He is much disposed to believe, that the affection is assumed, from the circumstance that the tremors are always most violent during the visits of the physicians in attendance. Under this impression, formed after one or two visits, he had prescribed a variety of the '*mistura diabolica*' of the military hospitals, which consists of a union of the tinctures of assafœtida, castor and aloes, with or without the aromatic spirit of ammonia, given repeatedly, so that the nauseous impression may be constantly on the tongue. In feigned cases of disease, the individual will rarely submit for any length of time to this disagreeable discipline; at the same time, it must be borne in mind, that it is upon the impression made on the gustatory nerves, and on those of the stomach, by "antispasmodics," that we depend principally in the treatment of hysteria; and hence, that the mistura diabolica, if successful, may not establish that the malady is assumed. Certainly benefit has resulted from the mixture.

Professor Dunglison next drew attention to some

### PATHOLOGICAL SPECIMENS.

The first was from a patient that died very soon after entering the hospital, and is interesting merely in its morbid relations, as no treatment was pursued in the case.

The patient, aged 59, was a man of very intemperate habits, and lived irregularly,—being dependent on the bounty of his neighbours for sustenance. He was so debilitated when he was received into the house, that no satisfactory examination could be made; but there was sufficient evidence of the existence of PNEUMONIA.

The lecturer observed, that his main object in exhibiting the specimen to the class was, to point out the appearances presented by a lung in the stage of purulent infiltration, or gray hepatization, which succeeds the stage of red hepatization. When this purulent secretion exists in pneumonia, the matter is rarely collected in abscesses, but is generally extensively diffused. It is uncommon, indeed, to find an abscess the consequence of inflammation of the pulmonary tissue.

The lung in this, as in all cases of a similar character, has lost its normal consistence, and has become exceedingly friable, so that the finger—as the lecturer showed—can be readily passed through it. [He here handed a portion of the lung to the class for examination.] In some parts of the lung—gentlemen would observe—there was also well marked, what has been termed the hyperæmic or congestive stage—a simple engorgement of the tissue, without any consolidation or breaking down of structure.

The left lung was almost entirely impermeable to air, and yielded a flat sound when percussed. The upper portion of the right lung was slightly affected, but the greater part of it was healthy. There were also slight adhesions between the pleura costalis and the pleura pulmonalis—the consequence of a previous inflammation of that membrane.

There was one other appearance to which the lecturer thought it proper to direct the attention of the class—as the physiology of the organ involved would soon form the subject of a lecture at the Jefferson Medical College—a diseased condition of the capsule of the spleen, without any alteration in the body of the organ. The glandiform body, in this case, was more lobated than natural, and its capsule was very much thickened, indicating that it must have been, at some time, the seat of active inflammation This inflammation, however, was manifestly not of recent date, and, consequently, could not have been the cause of death.

SPLENITIS is not an uncommon affection in malarious districts, in which the viscus, in consequence of the repeated congestions to which it is subjected, may become inflamed, or have its nutrition otherwise modified.

The diagnosis is not generally difficult. There is usually acute pain in the left hypochondrium, which is greatly increased by pressure, or motion of the part, and there may be, at the same time, if the spleen be engorged, dulness on percussion over a larger space than usual. The constitution, in acute cases, may be affected as in other cases of internal inflammation; but it rarely sympathizes to the same degree as in inflammation of more important organs. It may be confounded with peritonitis of the parietes of the abdomen; but as the treatment is the same, this would be of little importance. In the present instance, the capsule was principally affected, as the tissue of the spleen presents its normal appearance.

From a consideration of the *post-mortem* appearances, it is evident that death resulted in this case from pneumonia.

Tuberculosis of the Intestines.—This interesting specimen was obtained from a child 6 years old, who laboured also under scrofulosis of the spinal column. The lecturer did not see this case during life, as it occurred in another part of the establishment. The specimen was kindly brought to him by Dr. Wilson—one of the resident physicians of the hospital—under the well-founded idea, that the lecturer might consider it worthy of some pathological observations to the clinical class.

The whole of the peritoneal surface was shown to be completely studded with tubercles, presenting an excellent example of tubercular peritonitis. The inflammation, in this instance, must have been very extensive, although it was but little manifested during life. Whenever tubercles exist in any other part of the organism, they are generally found also in the lungs. In this case, the number there is very small.

In addition to the symptoms of caries of the spine, there were others of cerebral irritation, and it became a question, whether there might not be a tuberculous condition of the meninges of the brain, which is not unfrequent in scrofulous children; and has been shown by Papavoine, Fabre and Constant, Rufz, and by one of the lecturer's colleagues in this hospital—Dr. Gerhard—to be the anatomical character in many cases of acute hydrocephalus.

On examination, in this case, the encephalic meninges were not found diseased, and the cerebral irritation was, therefore, probably sympathetic.

The most remarkable feature of tuberculosis of the intestines or peritoneum proper is, that there are occasionally no very marked symptoms of its existence during life. It is, indeed, by no means an uncommon occurrence for death—as in the present case—to result from this cause, when its existence was scarcely or not at all suspected. The same remarks apply, indeed, to ordinary chronic peritonitis, which is often unmarked by pathognomonic phenomena.

The lecturer, by way of illustration, narrated a highly instructive case of a young lady in whom chronic inflammation of the peritoneum, producing distension of the abdomen, partly from purulent effusion, but mainly from tympanitis, was treated for ordinary ascites by one or two physicians, called in a few days only prior to dissolution.

There were, at the time the lecturer saw her, about two months before her death, no prominent manifestations of peritonitis. Attention, indeed, was mainly directed to the condition of the uterus and ovaries. The body, when placed upon the table, exhibited great abdominal distension; but on percussion, it was manifest that this was caused almost wholly by air. It was proposed, that a trocar should be first introduced, when, not a little to the astonishment of the physician last in attendance, the supposed dropsical effusion proved to be mainly a collection of air,—the abdomen immediately subsiding on its escape. Dissection exhibited that extensive chronic inflammation of the intestinal peritoneum had glued the folds of the intestines together, and that perforation of the intestines had occurred,—some of their contents, mixed with pus, being found in the cavity of the peritoneum. Such cases sufficiently show the latent character of this disease, and the consequent difficulty which must exist in the diagnosis. A similar latency is not unfrequent in chronic pleuritis.

Tubercles of the peritoneum may be either the cause or consequence of inflammation. In the vast majority of cases, they probably exist first, and, by their presence, excite fatal peritonitis.

In concluding, the lecturer begged the members of the class to examine these morbid specimens closely for themselves; reminding them that it was here, and in similar establishments only, that opportunities existed for pursuing, with full advantage, their researches in pathological anatomy.

